# Radiographic and tomographic findings of excretory urography of avian kidneys after induced nephropathy in broiler chicken as a model

**DOI:** 10.1002/vms3.70036

**Published:** 2024-11-19

**Authors:** Mahsa Zangisheh, Majid Masoudifard, Seyed Ahmad Madani, Alireza Vajhi, Hamideh hasannejhad, Ameneh Ghojoghi

**Affiliations:** ^1^ Department of Veterinary Surgery and Diagnostic Imaging, Faculty of Veterinary Medicine University of Tehran Tehran Iran; ^2^ Department of Animal and Poultry Health and Nutrition, Faculty of Veterinary Medicine University of Tehran Tehran Iran; ^3^ Department of Food Hygiene and Quality, Faculty of Veterinary Medicine University of Tehran Tehran Iran

**Keywords:** avian kidneys, broiler chicken, CT scan, excretory urography, nephropathy, radiography

## Abstract

**Background:**

Although kidney diseases have a high prevalence, initial recognition and diagnosis of renal disease are complicated in avian medicine.

**Objective:**

The aim of this study is to assess the intraosseous excretory urography (IOEU) in order to introduce a new method for evaluation of kidney's function in broilers as a primary model of birds.

**Methods:**

A total of 10 male broiler chickens at the age of 20 days were included and evaluated with plain and serial post‐contrast radiography and computed tomography (CT) scan to check the time and concentration of the contrast media (CM) passage from the kidney. In the next step, after the nephropathy induction with gentamicin, plain and serial post‐contrast radiography and CT scan were repeated. After the necropsy, kidney samples were sent for histopathology evaluation, and statistical analysis of data obtained from image analysis was conducted.

**Results:**

After nephropathy induction, all of the chickens exhibited increase in kidney size and in histopathological evaluation, all the samples (30 of 30) showed severe tubular necrosis and mild glomerulopathy. In the IOEU, the average time to create the maximum concentration of CM, the average time to decrease the CM concentration from the kidney and the time of the complete clearance of CM indicated a significant incensement after nephropathy compared to the time before it.

**Conclusion:**

Intraosseous excretory urography (IOEU) is a safe contrast imaging modality and can be useful in the assessment of the urinary system in chickens to check the changes in kidney size and function.

## Graphical abstract

Intraosseous excretory urography (IOEU) is a safe contrast imaging modality, and the use of radiography or CT scan is a useful method in the assessment of the urinary system in chickens.

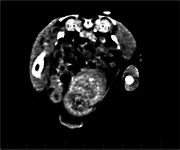

## 1 INTRODUCTION

Kidney diseases in birds are often secondary to other diseases and management disorders or can be associated with multisystemic conditions (Pollock, 2006). The diseases are classified as two main groups: primary diseases with a low prevalence and secondary diseases with a high prevalence (Altuzarra et al., 2018). Despite the high prevalence of kidney diseases in birds, combination of kidney diseases with symptoms of other conditions and lack of pathognomonic signs make it difficult to diagnose. Furthermore, birds have no specific biomarkers for kidney disease. Checking the blood uric acid level and creatinine is an insensitive marker in kidney diseases in birds; therefore, it cannot be considered a reliable diagnostic factor in kidney diseases (Canny, 1998; Harrison & Lightfoot, 2006; Hofbauer & Krautwald‐Junghanns, 1999; McMillan, 1988). Although initial recognition and diagnosis of renal disease is complicated in avian medicine, an operational method for the evaluation of kidney function could provide a good chance of treatment for patients (Pollock, 2006). Hence, in many cases, more than one diagnostic method is required to ensure renal involvement and differential diagnosis.

A kidney tissue biopsy provides the most information about kidney conditions among laboratory diagnostic methods and imaging; however, its use in the clinic is limited owing to its invasiveness and complications like bleeding after the biopsy (McMillan, 1988; Murray & Taylor, 1999; Suedmeyer & Bermudez, 1996). Diagnosis of a renal disorder requires multiple laboratory tests, complete clinical assessments and diagnostic imaging (Echols, 2006; Pollock, 2006). Clinical indications for using diagnostic imaging in kidney evaluation in birds include polydipsia, a long period of lack of access to water, poisoning, severe nutritional problems, polyuria, urine colour change, vomiting and dehydration (Echols, 2006; Krautwald‐Junghanns et al., 2008; Orosz & Toal, 1992; Veladiano et al., 2016).

The paired kidneys of birds have a specific anatomic location, located in the retroperitoneal space, on each side of the vertebral column, in a fossa ventral to the synsacrum. Each kidney has three lobes: cranial, middle and caudal (Krautwald‐Junghanns et al., 2008; Siller, 1981). Diagnostic imaging is one of the critical tools for evaluating the diseases of the urinary system in birds, playing a crucial role in avian medicine and exotic animals. It includes various methods such as plain radiograph, contrast media (CM) radiography, nuclear scintigraphy, magnetic resonance imaging and computed tomography (CT) scan. It is often clinically limited to simple methods such as plain radiography and ultrasound, so that other modalities are rarely used (Gumpenberger & Henninger, 2001; Krautwald et al., 1992; Krautwald‐Junghanns & Konicek, 2020; Pollock, 2006). Because kidneys are located in the synsacral fossa, a plain radiographic assessment of the avian kidney is challenging. In the ventrodorsal (VD) view, they are obscured by other parenchymal organs and are visible in the lateral view. In the lateral projection, an enlarged cranial renal division is the most reliable radiographic indicator of renomegaly. The kidney becomes visible in the VD view if its cranial renal division is enlarged (Krautwald et al., 1992; Simoes et al., 2012). Owing to the air sacs of the respiratory system, imaging the kidneys and ureters in healthy birds is typically impossible unless intracloacal probe is used. In the case of ascites and organomegaly, ultrasound can be used to evaluate the kidney parenchyma in birds due to air sacs compression (Bronneberg & Taverne, 2003; Schoemaker et al., 1997). Although the CT scan is typically used to detect musculoskeletal or respiratory problems, it can also be utilized to make or confirm a diagnosis of renal disease. The main benefit of CT is its three‐dimensional imaging evaluation without the superimposition of other structures. Owing to the presence of abdominal air sacs, the avian kidneys are clearly visible in CT scans (Krautwald‐Junghanns et al., 2008; Lierz, 2003).

CM‐containing iodine helps to diagnose some diseases in mammals and humans; however, it is infrequently used in avian medicine. The kidney and the ureter size, shape, position and function can be evaluated using excretory urography (IVEU). This technique is widely used in both human and veterinary medicine (Banner & Pollack, 1980; Fahmy & El‐habashi, 2015; Pollock, 2006). The impaired renal function may change the time of the presence of CM in the kidney or ureter. This technique can also assess the ureter function (e.g. after ureteroliths are surgically removed). Nevertheless, the absence of knowledge or experience in using this method in the kidneys evaluation of birds results in its limited use by clinicians (Banner & Pollack, 1980; Dennis & Bennett, 2000; Krautwald‐Junghanns & Tellhelm, 1994; Krautwald‐Junghanns et al., 1998).

Therefore, considering the high prevalence of kidney diseases, the difficult differential diagnosis with conventional methods and the critical role of diagnosis time in preventing systemic disorders, it is necessary to employ other diagnostic methods for evaluating the kidney function in order to accelerate the diagnosis and treatment. Accordingly, the present study aimed to use diagnostic imaging with the help of contrast material and to introduce new methods that could be used in the practice in order to evaluate kidney function in broilers as a primary model in the evaluation of all birds. This study helped to assess the use of intraosseous excretory urography (IOEU) in nephropathy‐induced birds in comparison to healthy birds to determine the times of beginning, maximum and end of the nephrographic phase via radiography and CT scan.

## 2 MATERIALS AND METHODS

### 2.1 Animals

In this study, 10 male broiler chickens were procured from a commercial poultry farm and then were kept in wire cages under standard management conditions. The chickens were 17 days old, had similar body weights and were free from any clinical signs.

### 2.2 Preparation

Ten, 17‐day‐old, male broiler chickens were included in this study. The chickens were acclimated in the Veterinary Teaching Hospital, Faculty of Veterinary Medicine, University of Tehran for 3 days to adaptation, under 12‐h dark/12‐h light cycles, humidity (55%–65%) and at constant temperature (25 ± 2°C). The chickens were provided with a commercial diet and sufficient water. This standard condition was implemented during the experiment.

### 2.3 Contrast media administration

After sedation (ketamine at dose rate of 2 mg/kg IM and medetomidine at the rate of 0.2 mg/kg IM) (Clarke & Trim, 2013), a non‐ionic iodine contrast agent (Iohexol) was used at a dose of 2 mL/kg in a solution with a concentration of 70%–80% of the contrast agent (300–400 mg ‏iodine/mL). In preparation for urography, the birds were fasted and hydrated, and the contrast medium containing iodine was warmed to body temperature and intraosseous injection was done. In order to injection, the intraosseous injection in the proximal cancellous bone of tibia was performed in this examination; after ensuring of aseptic condition, the needle was positioned at the flexion point of the chicken's knee joint, whereas the chicken was in lateral recumbency. The needle was inserted into the tibia medulla, the absence of resistance confirmed its placement in the medullary cavity and intraosseous injection was done (Yayla et al., 2018).

### 2.4 Radiography and CT‐scan examination before nephropathy induction

After sedation, the initial plain digital radiographic images of the celomic cavity were obtained in lateral and ventro‐dorsal positions using portable X‐ray tube unit (model SY‐HF‐110) and CR digital radiography (DirectView Kodak CR system) and exposure factors of 2.6 MAs and 50 kV. Subsequently, after tibial‐intraosseous CM injection, radiographs were immediately taken after the administration of the bolus, and at 1, 2, 5, 10, 20 min and more until the complete passage of CM from the urinary tract, using the same exposure factors and the same positioning as the survey study.

For clearance of the CM, the birds were rested for 24 h and then plain CT scan (using *Siemens Somatom Spirit* Dual Slice *CT‐scanner)* was performed, whereas the chickens were positioned in dorsal recumbency (MA 130, kV50, slice thickness 2 mm). Consequently, after the administration of CM, CT scan was immediately repeated at 1, 2, 5, 10, 20 min and more until the complete passage of CM from the urinary tract.

### 2.5 Nephropathy induction

To induce the nephropathy, gentamicin (gentamicin sulphate, 20% R Caspian‐tamin) with a dosage of 5 mg/kg intramuscularly every 12 h for 5 days (Fahmy & El‐habashi, 2015; Ijaz et al., 2008) was used from the age of 22 days (Banner & Pollack, 1980; Lierz, 2003; Lumeij, 2000).

### 2.6 Radiography and CT‐scan examination after nephropathy induction

After nephropathy induction, the process of plain‐ and post‐contrast radiography and CT scan was repeated for each bird according to the previous descriptions.

### 2.7 Image interpretation

Plain‐ and post‐contrast studies were performed and interpreted for renal size and opacification of the kidneys. The specific findings of IOEU included the beginning time of the nephrogram, the maximum CM concentration in kidney parenchyma, the time of decreased CM concentration and the complete clearance CM of the kidney. Finally, the difference in the size and the CM passage between the two groups were analysed.

Finally, after euthanizing with the increase dose of anaesthesia, kidney samples from each lobe (cranial, middle and caudal) were collected from the birds, and histopathological alternations of the kidney were evaluated using haematoxylin and eosin staining in the pathology laboratory.

### 2.8 Analytical statistics

Statistical analysis was conducted using the Statistical Package for the Social Sciences, Version 25 (IBM Corporation). Mean (±SD) was used to describe the samples’ characteristics in each group. Shapiro–Wilk test was used to check normal distribution, and the Paired *t*‐test and the Wilcoxon Signed Ranks test were used to compare the groups before and after the nephropathy induction. Statistical significance was defined as a *p*‐value less than or equal to 0.05 (Munro, 2005).

Because the chickens were in growing age, comparisons of kidney sizes were calculated on the basis of the ratio of kidneys length to the length of femoral bone by using the Chi Square test (Munro, 2005).

## 3 RESULTS

**FIGURE 1:**
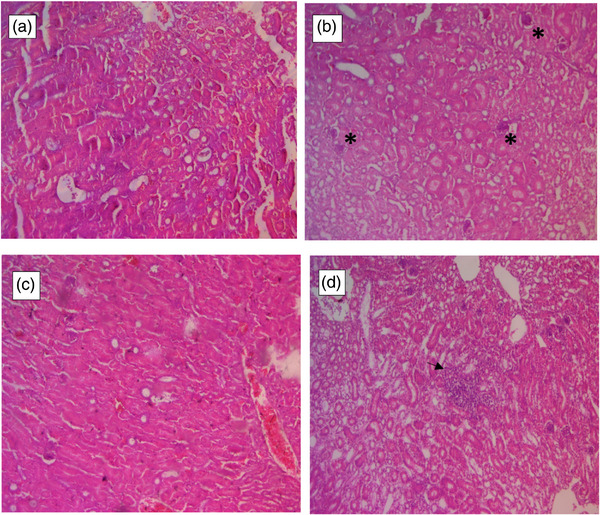
Micrographs of tubular changes in different kidney lobes after nephropathy induction. (a) Tubular necrosis in cranial lobe. (b) Tubular necrosis, tubular dilation and spheroid bodies (asterisks) are visible in middle lobe. (c) Severe tubular necrosis in distal lobe. (d) Tubular necrosis and lymphoid aggregation (arrow) in distal lobe. Haematoxylin and eosin (H&E)×10.

Pathology assessment of the renal samples, which was conducted blindly, confirmed the pathological changes in the kidney tissue after the nephropathy induction with gentamicin. According to the report from the 60 samples were prepared from all 3 kidney lobes, all the samples (30 of 30) exhibited severe tubular necrosis, 2 samples indicated severe inflammation, 27 samples had mild inflammation, and 1 sample lacked inflammation and except for one case, all the samples exhibited mild glomerulopathy (Figure 1).

### 3.1 The length of the kidney in radiography and CT scan

The comparison of proportion of kidneys length to the femoral bones was not significant (*p* > 0.05), but significant difference in kidney length between normal and nephropathy groups was due to nephropathy induction (*p* = 0.243 in radiographic examination and *p* = 0.227 in CT‐scan examination).

### 3.2 Digital radiography excretory urography

**FIGURE 2:**
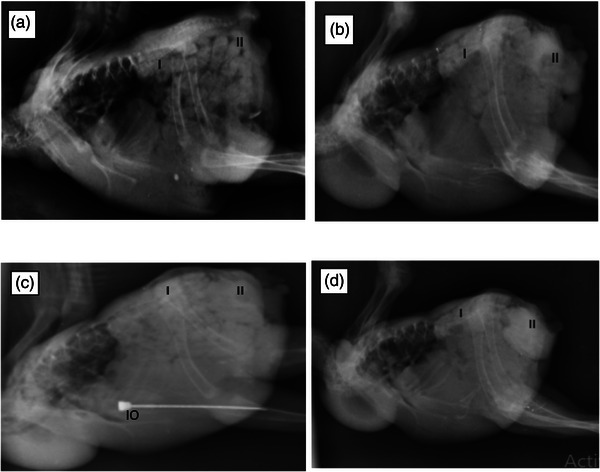
Plain‐ and post‐contrast radiography examination from normal kidney parenchyma (a, b) and after the nephropathy induction (c, d). In plain radiographs (a, c), normal opacity of the kidney is visible; in post‐contrast radiographs (b, d), contrast media (CM) results in the increased opacity of the kidney's parenchyma and coloac. Kidney (I), coloac (II), intraosseous needle (IO).

In the post‐contrast radiographic examination of the kidneys, the time of the CM entrance into the kidney parenchyma in both groups was 1 min after the injection. In this respect, no considerable distinction was found between the two groups (Figure 2).

In healthy chickens, the average time to create the maximum concentration of CM was 2.30 ± 0.48 min, and the average time to decrease the CM concentration from the kidney was 4.0 ± 80.42 min. The time of the complete clearance of CM was 8.1 ± 8.03 min after the injection, which increased to 4.0 ± 50.84, 10.80 ± 4.04 and 27.5 ± 7.54 min after the nephropathy induction, respectively (Table 1), indicating significant incensement compared to the time before the injection of gentamicin (*p *= 0.01a, *p *= 0.005a and *p* < 0.001b, respectively).

**TABLE 1:** Comparison of the time of entrance, persistence and clearance of the contrast media (CM) in the radiography of chickens before and after the nephropathy induction.

Variable		Mean ± SD	*Z*/*t*	*p*‐Value
Maximum CM concentration in RD^c^	Normal kidney	2.30 ± 0.48	*Z* = 2.53	*p* = 0.01^a^
	Nephropathy kidney	4.50 ± 0.84		
Decreased CM concentration in RD	Normal kidney	4.80 ± 0.42	*Z* = 2.80	*p* = 0.005^a^
	Nephropathy kidney	10.80 ± 4.04		
Complete clearance of CM in RD	Normal kidney	8.80 ± 1.03	*Z* = 2.80	*p* < 0.001^b^
	Nephropathy kidney	27.50 ± 7.54		

^a^ Wilcoxon signed rank test.

^b^ Paired *t*‐test.

^c^ Radiography.

### 3.3 CT‐scan excretory urography

**FIGURE 3:**
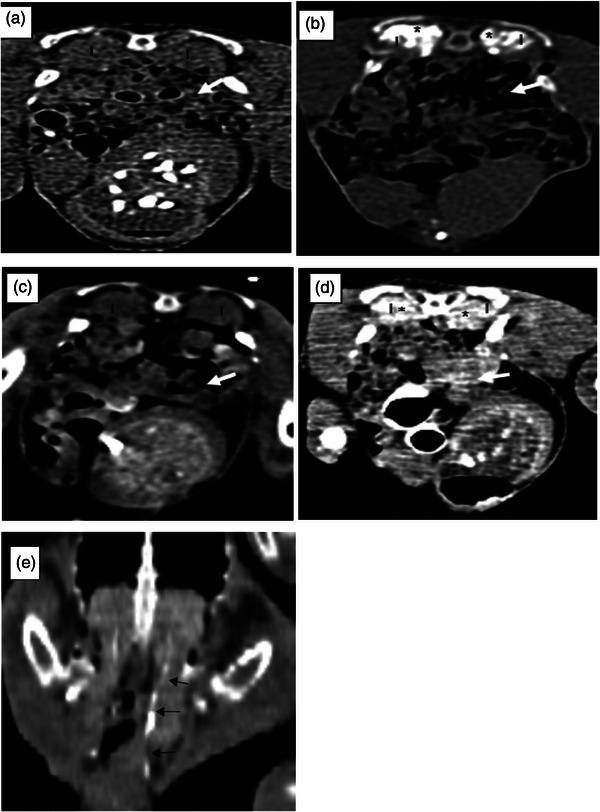
Plain‐ and post‐contrast computed tomography (CT)‐scan examination from normal kidney parenchyma (a, b) and after the nephropathy induction (c, d). In the plain CT scan (a, c), normal opacity of the kidney is visible; in the post‐contrast CT scan (b, d), contrast media (CM) results in the increased opacity of the kidney parenchyma (asterisks) and (e) coronal plane of post‐contras CT scan which shows passage of CM from ureter (arrow). Kidney (1), GI tract (arrow).

In the comparison, which was performed to examine the kidneys of normal birds and nephropathy‐induced birds via post‐contrast CT scan, the time of the CM entrance into the kidney in both groups was at the first minute after the injection (Figure 3). However, it showed a significant increase in the time of creating the maximum CM concentration (2.0 ± 70.67 and 5.00 ± 1.82), the time of decreasing concentration (4.80 ± 0.63 and 9.80 ± 1.61) and the time of complete clearance of the contrast agent from the kidney (7.70 ± 0.67 and 24.00 ± 5.16) in the control and nephropathy‐induced birds, respectively (Table 2).

**TABLE 2:** Comparison of the time of entrance, persistence and clearance of the contrast media (CM) in the computed tomography (CT scan) of chickens before and after the nephropathy induction.

Variable		Mean ± SD	*Z*/*t*	*p*‐Value
Maximum CM concentration in CT scan^b^	Normal kidney	2.70 ± 0.67	*Z* = 2.32	*p *= 0.020^a^
	Nephropathy kidney	5.00 ± 1.82		
Decreased CM concentration in CT scan	Normal kidney	4.80 ± 0.63	*Z* = 2.81	*p *= 0.005^a^
	Nephropathy kidney	9.80 ± 1.61		
Complete clearance of CM in CT scan	Normal kidney	7.70 ± 0.67	*Z* = 2.81	*p* = 0.005^a^
	Nephropathy kidney	24.00 ± 5.16		

^a^ Wilcoxon signed rank test.

^b^ Computed Tomography scan.

## 4 DISCUSSION

In this study, owing to the renal portal system in birds, the contrast agent is quickly secreted if the femoral vein is used. However, the jugular, medial metatarsal vein and ulnar vein can also be used without losing the test quality. It should be noted that intravascular injection could be complex due to small peripheral vessels in birds or their collapse. In addition to intravascular injection, an intraosseous injection can be used in birds. This method is preferable due to easier access and more excellent stability, which is easier for the bird to bear it (Dennis & Bennett, 2000; McMillan, 1988). For this reason, the intraosseous injection can be a suitable alternative to contrast agent injection or catheter placement. Thus, the intraosseous injection in the proximal cancellous bone of tibia was preferred in this examination.

In our study, the CM passage from the chicken's kidney parenchyma was evaluated, with radiography and CT scan, before and after the nephropathy induction to determine the difference in size of kidneys and states of nephrogram between the two groups. ntraosseous excretory urography (IOEU) appears to be a secure contrast imaging modality and can be useful in the assessment of the urinary system in chickens. This technique permits the examination the morphological features of the kidney and ureters as well as the assessment and determination of the time at which urographic phases occur to check the renal function (Krautwald‐Junghanns & Konicek, 2020).

IVEU has been a practical imaging method used in the assessment of human and other mammals due to qualification of the kidneys function. The findings in this examination were evaluated on the basis of the number, size, shape, location and density of the kidneys and urinary tracts, as well as according to the degree and time of changes in the CM density in nephrogram and pyelogram phases (Altuzarra et al., 2018; Armbrust & Grauer, 2015; Banner & Pollack, 1980; Feeney et al., 1982), despite the anatomical diversity of the urinary system between the mammals and the birds. The findings are compatible with our study observations in birds.

In this examination, features of the urogram phases were reviewed by using digital radiography in control group. In congruency with past studies, the vascular phase was immediately detectable after the beginning of the intraosseous contrast administration. Beginning of the nephrogram phase was visible at 1 min after contrast administration. The maximum concentration of CM, beginning of decrease CM opacity of kidney's parenchyma and complete clearance were identified at about the time of 2.5, 5 and 8 min after CM administration, respectively. This finding is compatible with previous examinations that used iodine compounds to check kidney function in normal birds; CM was observed in the heart and pulmonary vessels 10 s after injection, in the kidney 50–20 s later and after 5–2 min it entered the cloaca (Dennis & Bennett, 2000; Krautwald‐Junghanns et al., 2008; Pollock, 2006).

In our examination, the CM passage in the urinary tract was also evaluated using CT‐scan examination, indicating the same results with the assessment using the same protocol with radiography; however, CT scan is more sensitive, because the evaluation of the entire urinary tract is possible accurately and noninvasively without the superimposition of other organs.

A complex interaction of renal perfusion, normal glomerular filtration, tubular function and the absence of urinary tract obstruction is required for normal clearance of CM from the kidney. Alterations to these factors may result in both quantitative and qualitative IVEU or IOEU image abnormalities. Numerous abnormal patterns of nephrographic opacification and fade‐out have been defined in human and veterinary publications and correlated with various pathologies (Feeney et al., 1982; Simoes et al., 2012). Furthermore, acute renal diseases, conditions affecting the general renal function and obstructive disease all create changes in passage time and concentration of CM from the kidney (Armbrust & Grauer, 2015; Krautwald‐Junghanns et al., 2008; Murray & Taylor, 1999), being consistent with our findings from IOEU with CT scan and radiography in chickens after the nephropathy indication in which the beginning of the excretory phases was detected at the same time (at the first minute after injection); but detecting of maximum CM concentration, beginning decrease opacity and complete clearance of CM form kidney's parenchyma took more time than control group (mean times are, respectively: 5, 10 and 25.5 min). Previous examinations in mammals also showed that inadequate renal function might result in much slower elimination of the contrast agent, resulting in delayed ureter filling (Banner & Pollack, 1980). Therefore, prolonged elimination times indicate renal insufficiency.

Recent studies have demonstrated that obstructive diseases are accompanied by changes in nephrographic opacification in IVEU, and this method can also help to find the obstruction site in ureter and post operation evaluations in both mammals and avian classes; the method was used in an Amazon parrot to check the peristaltic movements and urethral size (Dennis & Bennett, 2000).

Similar to many of the previous examinations, in this study, the kidneys exhibited significant increase in size of renal divisions that is visible in lateral view of radiographic examination as bulging of cranial kidney pole and is measurable in CT scan as increase in kidney size.

Gentamicin is commonly used in birds; however, it has nephropathy effects that result in kidney enlargement and changes resembling other causes of renal failure. Histopathological finding of our study indicated that all of the samples showed severe tubular necrosis, 2 samples indicated severe congestion, 27 cases mild inflammation and except for one case, all samples showed mild glomerulopathy. These findings are compatible with previous histopathological findings that revealed marked tubular vacuolar degeneration (Fahmi & El‐habashi, 2015; Khan et al., 2008); focal interstitial mononuclear cell infiltrations, congestion, focal cystic change and tubular necrosis in Gentamicin‐treated kidneys. Hence, the cause of this discrepancy is related to an acute disturbance in the function of glomerular infiltration and tubular resorption after the nephropathy induction. Therefore, in birds suffering from glomerular or tubular nephropathy due to kidney dysfunction, renal filtration is affected, affecting the passage of CM in different phases in the kidney. Thus, it can be used in diagnosing and confirming kidney diseases with the help of diagnostic imaging (Fahmi & El‐habashi, 2015; Khan et al., 2008).

This study reveals that excretory urography in both CT‐scan and radiography methods provides a clear image of the kidneys and indicates that IOEU can play a key role in diagnostic procedures of the urinary system in chickens as a model. Moreover, intravenous CM injection is contraindicated in patients with a history of an adverse reaction to iodinated contrast, dehydration and severe renal impairments. Consequently, to prevent these complications, free access to water was allowed to ensure proper hydration (Heuter, 2005).

The standard and nephropathy characterizations of IOEU described in this study will provide the veterinarian a valuable tool to evaluate renal function and morphologic kidney features. The results of this study can be used as a primary reference value in the birds; however, the standardization of protocols for IOEU is required to facilitate the comparison of the patient results. Moreover, further research is necessary to evaluate the diagnostic efficiency of IOEU in other species of birds with kidney or ureteral disease.

It is essential to mention that the accessibility of radiography, the temporal limitation of the radiographic technique and its lower spatial resolution compared to CT scan may be crucial selection criteria for excretory urography.

## 5 CONCLUSION

To our knowledge, this is the first study evaluating and comparing the passage of CM in normal and nephropathy‐induced kidneys in chickens. Pathology assessment of the renal samples confirmed nephropathy changes following nephropathy‐induction. Additionally, Intraosseous excretory urography (IOEU) using both radiography and CT‐scan methods revealed key findings. First, it indicated an increase in the average time to create the maximum concentration of CM, the average time to decrease the CM concentration from the kidney and the time of the complete clearance of CM from kidney parenchyma. Second, the examination showed a noticeable increase in kidney size after nephropathy compared to the normal state.

## AUTHOR CONTRIBUTIONS

**Mahsa Zangisheh**: Investigation; project administration; writing – original draft. **Majid Masoudifard and Seyed Ahmad Madani**: Supervision; project administration. **Alireza Vajhi: Supervision and Ameneh Ghojoghi**: Investigation. **Hamideh hassannejhad**: Validation.

## CONFLICT OF INTEREST STATEMENT

The authors declare no conflicts of interest.

## FUNDING INFORMATION

None.

## ETHICS STATEMENT

IR.UT.VETMED.RED.1401.015 (The authors confirm that the ethical policies of the journal, as noted on the journal's author guidelines page, have been adhered to. No ethical approval was required. None were injured.)

### PEER REVIEW

The peer review history for this article is available at https://publons.com/publon/10.1002/vms3.70036

## Data Availability

Data is available within the article.
